# Age, income, and the discounting of delayed and probabilistic rewards

**DOI:** 10.3389/fpsyg.2026.1765142

**Published:** 2026-06-12

**Authors:** Haoran Wan, Joel Myerson, Leonard Green, Michael J Strube, Sandra Hale

**Affiliations:** Department of Psychological & Brain Sciences, Washington University in St. Louis, St. Louis, MO, United States

**Keywords:** age, buffering hypothesis, discounting, income, intertemporal choice, psychological distress, risky choice

## Abstract

Recent meta-analyses of age-related changes in decision-making have yielded inconsistent findings, reporting no sizable age effect on intertemporal and risky choice (i.e., delay and probability discounting, respectively). However, these reviews did not systematically account for socioeconomic status, a potential moderator. The present research addresses this gap by examining the interactive effects of age and self-reported annual household income on both intertemporal and risky choice across a wide range of reward amounts. In two parallel studies, participants aged 20 to 80 (Study 1, delay discounting: *N* = 596; Study 2, probability discounting: *N* = 592) completed an adjusting-amount discounting task with hypothetical rewards of $150, $2,500, and $30,000. As expected, income was unrelated to discounting in participants under 35. For those 35 and older, the effects of age were domain-specific: Degree of delay discounting decreased with age, as indicated by linear increases in the Area-under-the-Curve (AuC), whereas for risky choice, the relation between AuC and age was nonlinear, with discounting decreasing until around age 60 and relatively little change thereafter. As predicted by the buffering hypothesis, these age effects were moderated by income in both domains. For delay discounting, the age-related decrease in degree of discounting was present only in lower- and middle-income groups. For probability discounting, the nonlinear age trajectory was absent in the high-income group. These findings resolve prior inconsistencies by demonstrating that the effects of aging on economic preferences follow distinct, domain-specific trajectories that are markedly influenced by socioeconomic context.

## 1 Introduction

Many important everyday decisions involve trade-offs between the magnitude of an outcome and its delay or probability of occurrence. When the choice is between an immediate and a delayed reward, the subjective value of the delayed option decreases as the delay to its receipt increases, a phenomenon known as delay discounting; similarly, when the choice is between a certain and a probabilistic reward, the subjective value of the probabilistic option decreases as the odds against its receipt increase, a phenomenon known as probability discounting (for reviews, see Green and Myerson, 2004, 2018). Individual differences in degree of discounting are associated with differences in a variety of real-world decisions. Steeper discounting, for example, has been linked to adverse financial and health-related choices (Meier and Sprenger, 2012; Huffman et al., 2019) and a range of problematic behaviors, including substance abuse (Baker et al., 2003; Reynolds et al., 2004) and pathological gambling (Dixon et al., 2003; Holt et al., 2003). The degree of delay discounting has thus been proposed as a “transdiagnostic” marker that is associated with individual differences in a variety of problem behaviors and psychiatric disorders (Bickel et al., 2014; Amlung et al., 2016).

The existing literature on age-related changes in discounting has yielded inconsistent results, with some studies reporting increases in discounting with age (Kirby et al., 2002; Read and Read, 2004), others reporting decreases (Eppinger et al., 2012; Green et al., 1994; Reimers et al., 2009), while yet others failing to observe any significant age differences (Samanez-Larkin et al., 2011; Seaman et al., 2018). This is unfortunate because older adults often face high-stakes financial and health decisions involving delayed and/or probabilistic rewards. With regard to delay discounting, two recent meta-analyses reported that although older adults tended to discount delayed gains less steeply than younger adults, the difference was not “sizeable” (Seaman et al., 2022), with one referring to the effect as of “trivial” practical significance (Bagaïni et al., 2023). These findings challenge theories such as Socioemotional Selectivity Theory (Carstensen, 2021) that predict steeper discounting with age. The literature on decision-making involving probabilistic outcomes reveals a similar lack of a clear consensus. While probability discounting is one operationalization of valuation under risk, broader meta-analyses of risk preference between younger and older adults have failed to find a significant overall age effect (Mata et al., 2011; Best and Charness, 2015; Bagaïni et al., 2023). However, when isolating choices between certain and probabilistic gains – a paradigm structurally analogous to that of probability discounting – results indicate that younger adults choose the probabilistic option more often than older adults (Best and Charness, 2015; Bagaïni et al., 2023). This may indicate that age-related differences in choice behavior are specific to the valuation of probabilistic gains, rather than reflecting a generalized shift in risk tolerance.

A critical limitation of these meta-analytic reviews is their failure to evaluate the role of socioeconomic status. This omission is unfortunate because income has been identified as an important moderator of the degree of discounting (e.g., Green et al., 1996; Burro et al., 2022; Wan et al., 2024). Indeed, some of the inconsistencies reported in the aging literature may reflect the income differences between samples of younger and older adults. For example, a recent study found that income moderates the effect of age: Lower-income older adults discounted delayed rewards significantly less steeply than lower-income younger adults, but the age difference was not significant when higher-income samples were compared (Wan et al., 2024).

Wan et al. (2024) proposed a buffering hypothesis to explain the modulatory effect of income that they observed on the size of the age-related difference in delay discounting. Their hypothesis posits that while financial scarcity is a psychological stressor that leads to steeper delay discounting, there are age-related increases in emotional stability (Terracciano et al., 2005; Costa et al., 2019) that buffer older adults against various stressors, including financial scarcity. Thus, the age-related increase in emotional stability should mitigate the effects of scarcity-induced stress on decision-making. Specifically, it predicts that although financial scarcity (i.e., a low income) will lead to steep discounting in younger adults, it will have less of an effect on older adults, leading to a significant age difference. In contrast, the absence of financial scarcity (i.e., a high income) is not associated with psychological distress, and therefore high-income younger and older adults will both show shallower discounting, and the size of the age-related difference will decrease. Indeed, in a direct test of this hypothesis, Wan et al. confirmed the predicted pattern. That is, the age difference observed in the lower-income groups was eliminated after statistically controlling for psychological distress.

The domain-generality of the modulatory effect of income on age-related differences, however, remains unknown. Whether, and how, the buffering hypothesis applies to risky choice is an important question. Indeed, Mejía-Cruz et al. (2016) examined the discounting of delayed and probabilistic outcomes and found that delay and probability discounting of various kinds of rewards loaded on two separate factors. Moreover, as in previous studies, amount had opposite effects on the discounting of delayed and probabilistic rewards: Whereas larger delayed rewards were discounted less steeply than smaller delayed rewards, larger probabilistic rewards were discounted more steeply than smaller probabilistic rewards (Myerson et al., 2011; Green et al., 2014a). Given that delay and probability discounting of rewards appear to represent separate constructs, it remains an open question whether the moderating role of income on age differences in discounting extends to risky choice. Accordingly, the present investigation provides the first systematic examination of the interactive effects of age and income on risky choices, as well as a comparison of the effects of income and age on delay discounting and probability discounting.

The present investigation also examined the effect of reward amount on age-related differences in decision-making. A meta-analysis by Best and Charness (2015) revealed that age-related decreases in probability discounting are more pronounced for small monetary amounts. They reported that younger adults were significantly more risk-seeking than older adults when the amounts were small, but that this age difference disappeared for large amounts. Similarly, Wan et al. (2024) also observed larger age-related differences with smaller delayed reward amounts, although the largest amount studied was $500. To address limitations of previous research that often used relatively small monetary amounts (e.g., Reimers et al., 2009; Samanez-Larkin et al., 2011; Seaman et al., 2018), the present studies of delay and probability discounting employed a wide range of hypothetical amounts ($150, $2,500, and $30,000) with participants ranging from 20 to 80 years of age and representing a wide spectrum of household incomes (from <$30,000 to >$100,000 annual household income).

Finally, because the self-reported income of young adults may not be a reliable indicator of their experienced financial scarcity due to parental financial support (US Census Bureau, 2023), we first hypothesized that for participants under the age of 35, reported income would be unrelated to their degree of discounting. Our primary analyses, therefore, focused on participants aged 35 and older. Study 1 was a large-sample examination of delay discounting in three income groups testing whether, as our previous finding (Wan et al., 2024) suggested, age-related decreases in discounting would be present in lower-income groups but absent in a higher-income group. Study 2 tested whether our findings regarding income effects and delay discounting generalized to probability discounting. We hypothesized that older adults would exhibit greater risk aversion (i.e., steeper probability discounting), while also seeking to characterize this age-related change by examining both linear and nonlinear relations and testing whether the buffering hypothesis would extend to risky choice. By examining these factors across two fundamental decision-making domains, this research aims to resolve inconsistencies in the literature on age and decision-making, highlighting the critical role of socioeconomic context.

## 2 General method

### 2.1 Participants

Separate samples were recruited for Study 1 and Study 2 from the pool maintained by Eyes4Research (an online platform for recruiting participants of various backgrounds and demographics) and screened for a U. S. IP address. Participants were recruited until the number in each of the six age decades (20–29, 30–39, 40–49, 50–59, 60–69, and 70–80 years of age) was about 100 after eliminating any who failed an attention check, with the constraint that the number of male and female participants in each decade be approximately equal.

### 2.2 Procedure

The study was approved by the Washington University in St. Louis Institutional Review Board, IRB No. 201806131, “Decision-making in younger and older adults: A discounting framework.”

After agreeing to participate in the study, participants in both studies completed an adjusting-amount discounting task (Du et al., 2002; Estle et al., 2006) consisting of three amount conditions: $150 (small), $2,500 (medium), and $30,000 (large); the order of the three amount conditions was randomly determined for each participant. After completing the adjusting-amount task, participants were administered the Hospital Anxiety and Depression Scale (HADS), which measures state anxiety and depression (Zigmond and Snaith, 1983), followed by a series of demographic questions and a question about their general health.

### 2.3 Measures

Discounting was measured at the individual level as the area under the empirical discounting curve (AuC; Myerson et al., 2001) calculated for each amount. The AuC is a widely used measure of discounting that, unlike other prominent measures (e.g., the rate parameter, *k*, of a hyperbolic function), is theoretically neutral and makes no assumptions regarding the mathematical form (e.g., exponential, hyperbolic, hyperboloid) of the relation between immediacy/certainty and the subjective value of a delayed/probabilistic reward. It is to be noted that AuC is inversely related to degree of discounting: A larger AuC indicates shallower discounting (i.e., the delayed or probabilistic reward retains greater subjective value), whereas a smaller AuC denotes steeper discounting.

Demographic variables included age (in years), gender (0 = male; 1 = female), annual household income (1 = <$30,000, 2 = $30,000–$50,000, 3 = $50,000–$80,000, 4 = $80,000–$100,000, 5 = > $100,000), years of education (5 = some grade school, 12 = high school graduate or G. E. D., 14 = some college, 16 = college graduate, 18 = any post-graduate or professional school). Psychological distress was assessed using the Hospital Anxiety and Depression Scale (HADS), which consists of seven questions each for anxiety and depression; possible scores on each scale ranged from 0 to 21 (total 0 to 42). The test–retest reliability and the internal consistencies of the two subscales are all high, and importantly, factorial analyses indicate that the subscales can be conceptualized as two theoretically independent dimensions (for a review, see Herrmann, 1997). Moreover, the HADS has been shown to be suitable for online administration (Andersson et al., 2003). General health was assessed with a single question, “At the present time, my overall health is?”, to which participants responded by selecting one of five categories: excellent, very good, good, fair, and poor, with “excellent” being 5 and “poor” being 1.

### 2.4 Analysis

For each study, our analyses began by establishing the representativeness of the discounting data. To that end, we examined whether the results reflected systematic decreases in the subjective value of the delayed or probabilistic reward. The mean relative subjective values (*RSV*) of the delayed or probabilistic reward (i.e., the subjective values divided by the actual amount) at each delay or odds against (*X*) in each amount condition were modeled with a hyperboloid function (Myerson and Green, 1995; Green et al., 1999) that typically describes discounting data (i.e., a hyperbola-like equation in which the denominator is raised to a power: *RSV* = 1/[1 + *bX*]*^s^*). For the purpose of fitting hyperboloid discounting functions, the participants were divided into four age groups (20–34, 35–50, 51–64, and 65–80 years).

For each study, we then examined the correlation between age and AuC for all participants (aged 20–80). To test our hypothesis of the effects of age and income on the discounting by those under 35 and those older, two separate correlational analyses between income and mean AuC were conducted: one for participants aged 20–34, and the other for participants aged 35 and older. Further analyses focused on the participants aged 35–80 and examined the effects of age and income on discounting, after which we tested models that also included distress, anxiety, or depression.

To test if there was an effect of age on discounting at some income levels but not others, linear contrasts of the age effect based on multilevel beta regression were examined at three levels of income: <$50,000, $50,000–$80,000, and >$80,000, controlling other variables at the mean level. Note that <$50,000 represents approximately the lower third of the national income distribution in 2022, and <$80,000 represents a little more than half of the distribution (actual median = $74,580; Guzman and Kollar, 2024). Linear contrasts were used to control for the lack of power for detecting ordinal interactions (Rosenthal and Rosnow, 1985; Strube and Bobko, 1989).

Multilevel analyses were used to account for the multiple amount conditions within individuals (i.e., three observations for each individual). The AuC measures were analyzed via beta regression because the AuC is continuous but restricted to the interval between 0 and 1 (Douma and Weedon, 2019; Wan et al., 2023). For all regression analyses, the logarithm of amount was used because of the range and skewed distribution of the reward amounts (i.e., $150, $2,500, and $30,000). All variables were centered at the grand mean and scaled by dividing them by two standard deviations, except for gender, which was only grand-mean centered as it is a binary variable.

After visual inspection of simulation-based diagnostic plots that compare the empirical cumulative distribution of simulated outcomes to the observed values, it was determined that the residuals deviated from the distribution expected in a properly specified beta regression model. To provide more defensible inferences, we employed non-parametric bootstrapping procedures using level 2 resampling with 5,000 samples. Each bootstrapped sample was constrained such that the number of participants reflected that of the original sample.

Major analyses were conducted using R Version 4.2.1 (R Core Team, 2022). The glmmTMB software package (Brooks et al., 2017) was used to conduct multilevel beta regression analyses, and the DHARMa software package (Hartig, 2024) was used to perform simulation-based residual diagnostics. In the Results section of each study, inferences were drawn from the median and standard deviation of the bootstrapped distribution, along with the corresponding *p*-value.

## 3 Study 1: delay discounting

### 3.1 Method

#### 3.1.1 Participants

A sample of 861 participants was recruited for Study 1. Of these, 257 failed at least one of the six attention checks presented during the discounting part of the study (e.g., “Please choose Lion”, and the choice options were “Lion” and “Elephant”; “Which would you prefer to receive? $150 Now or $75 Now?”), and eight selected “non-binary” in response to the question regarding gender. Statistical analyses were based on the remaining 596 participants (see Table 1 for demographic information).

**Table 1:** Characteristics of participants by age group in Study 1.

Characteristics	Age group			
	20–34 (*N* = 144)	35–50 (*N* = 154)	51–64 (*N* = 140)	65–80 (*N* = 158)
Age (years), Mean (SD)	27.06 (4.41)	41.81 (4.54)	57.21 (4.05)	71.35 (3.91)
Female, % (*N*)	50.00 (72)	51.30 (79)	50.71 (71)	49.37 (78)
Education (years), mean (SD)	13.98 (2.84)	14.46 (2.72)	14.23 (2.11)	14.60 (2.13)
(*N* Missing)	(1)	(0)	(1)	(0)
HADS				
Anxiety, mean (SD)	10.73 (4.69)	9.81 (5.16)	7.55 (4.30)	5.77 (4.35)
Depression, mean (SD)	6.76 (3.74)	6.70 (4.28)	5.74 (3.66)	4.98 (3.96)
(*N* Missing)	(4)	(2)	(1)	(2)
Health, mean (SD)	3.48 (1.07)	3.26 (1.11)	3.06 (0.93)	3.14 (0.94)
(*N* Missing)	(0)	(2)	(0)	(0)
Not Hispanic/Latino, % (*N*)	85.31 (122)	88.24 (135)	94.24 (131)	95.57 (151)
(*N* Missing)	(1)	(1)	(1)	(0)
Race, % (*N*)				
American Indian or Alaska Native	1.44 (2)	0.65 (1)	0.72 (1)	0
Asian or Asian American	6.47 (9)	1.31 (2)	0	0
Black or African American	19.42 (27)	9.80 (15)	10.14 (14)	6.33 (10)
Native Hawaiian or Pacific Islander	0.72 (1)	0.65 (1)	0	0
White	67.63 (94)	81.70 (125)	86.96 (120)	92.41 (146)
More than one race	4.32 (6)	5.88 (9)	2.17 (3)	1.27 (2)
(*N* Missing)	(5)	(1)	(2)	(0)
Income (in U. S. dollars), % (*N*)				
<$30,000	30.37 (41)	32.39 (46)	29.63 (40)	31.33 (47)
$30,000–$50,000	22.96 (31)	15.49 (22)	30.37 (41)	25.33 (38)
$50,000–$80,000	22.22 (30)	22.54 (32)	20.74 (28)	26.00 (39)
$80,000–$100,000	11.11 (15)	7.04 (10)	8.15 (11)	11.33 (17)
>$100,000	13.33 (18)	22.54 (32)	11.11 (15)	6.00 (9)
(*N* Missing)	(9)	(12)	(5)	(8)

SD = standard deviation; N = number of participants; HADS = Hospital Anxiety and Depression Scale.

#### 3.1.2 Procedure

Within each of the three amount conditions, each participant made a series of six choices at each of five delays (1 month, 3 months, 1 year, 3 years, and 10 years) between a smaller, immediate reward, whose amount was adjusted, and a larger, delayed reward, whose amount remained constant within a condition. On each trial, if the participant chose the smaller, immediate reward, its amount was decreased on the next trial, whereas if the participant chose the larger, delayed reward, the amount of the immediate reward was increased on the next trial. The first choice in each delay condition was between the delayed reward and an immediate reward whose amount was half that of the delayed reward, and the first adjustment was half of the difference between the amounts of immediate and delayed rewards; the size of each subsequent adjustment was half that of the previous adjustment, rounded to the nearest dollar. For example, in the condition where the delayed reward was $2,500 to be received in a year, the choice on the first trial would be between receiving $2,500 in a year and $1,250 right now. If the participant chose to receive $2,500 in a year, then the choice on the second trial would be between $2,500 in a year and $1,875 right now. If the participant then chose to receive $1,875 right now, the choice on the third trial would be between receiving $2,500 in a year and receiving $1,563 right now.

The adjusting-amount task converges on an amount of immediate reward that approximates the subjective (present) value of the delayed reward. The amount of the immediate reward that would have been presented on a seventh trial, if one had been presented, provides an estimate of the subjective value of the delayed reward. The procedure was repeated at each of five delays in order to map out a discounting function reflecting the change in the subjective value of the reward as the delay to its receipt increased. Within each of the three amount conditions, the order of presentation of the five delay conditions was randomly determined.

### 3.2 Results

**Figure 1:**
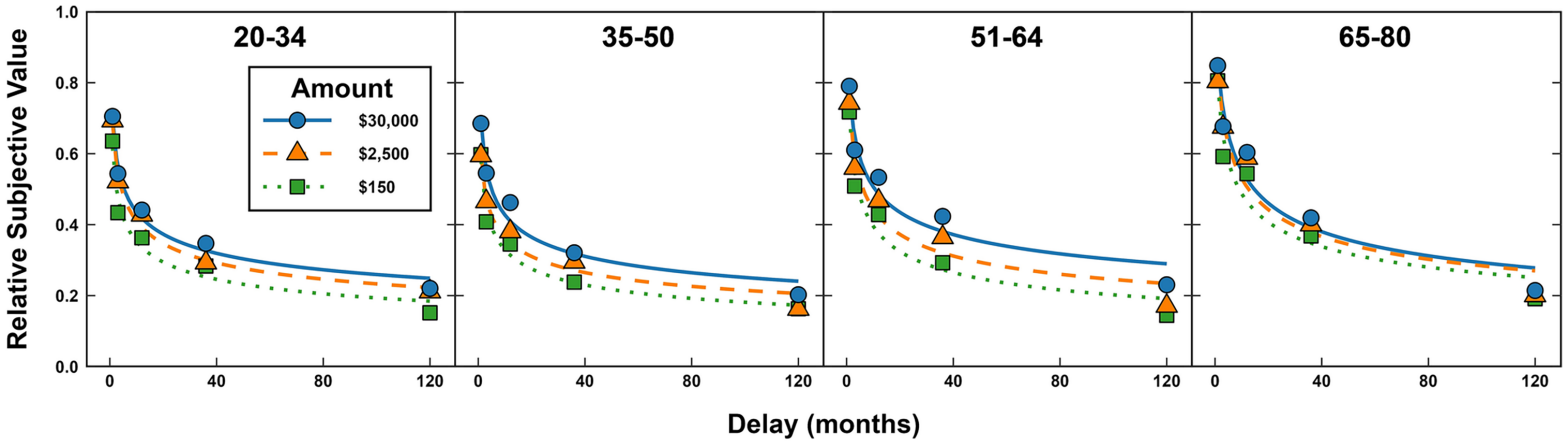
Relative subjective value of a reward as a function of the time until its receipt for each reward amount for each age group in Study 1. Data represent the group means, and curves represent the best-fitting hyperboloid discounting functions.

Figure 1 depicts the group mean relative subjective values of the delayed reward (i.e., subjective value divided by the delayed amount) as a function of the delay in each amount condition for each age group. As may be seen, typical discounting was observed in all four age groups: Relative subjective value decreased systematically as a function of delay in each of the three amount conditions, and in each case, the hyperboloid discounting function (Myerson and Green, 1995) accounted for more than 93% of the variance. Notably, a clear amount effect was observed: Larger delayed amounts were discounted less steeply than smaller delayed amounts. For all four age groups, linear contrasts based on beta regressions verified that there was a systematic increase in AuC as a function of the size of the delayed amount (all *p* < 0.003).

**Figure 2:**
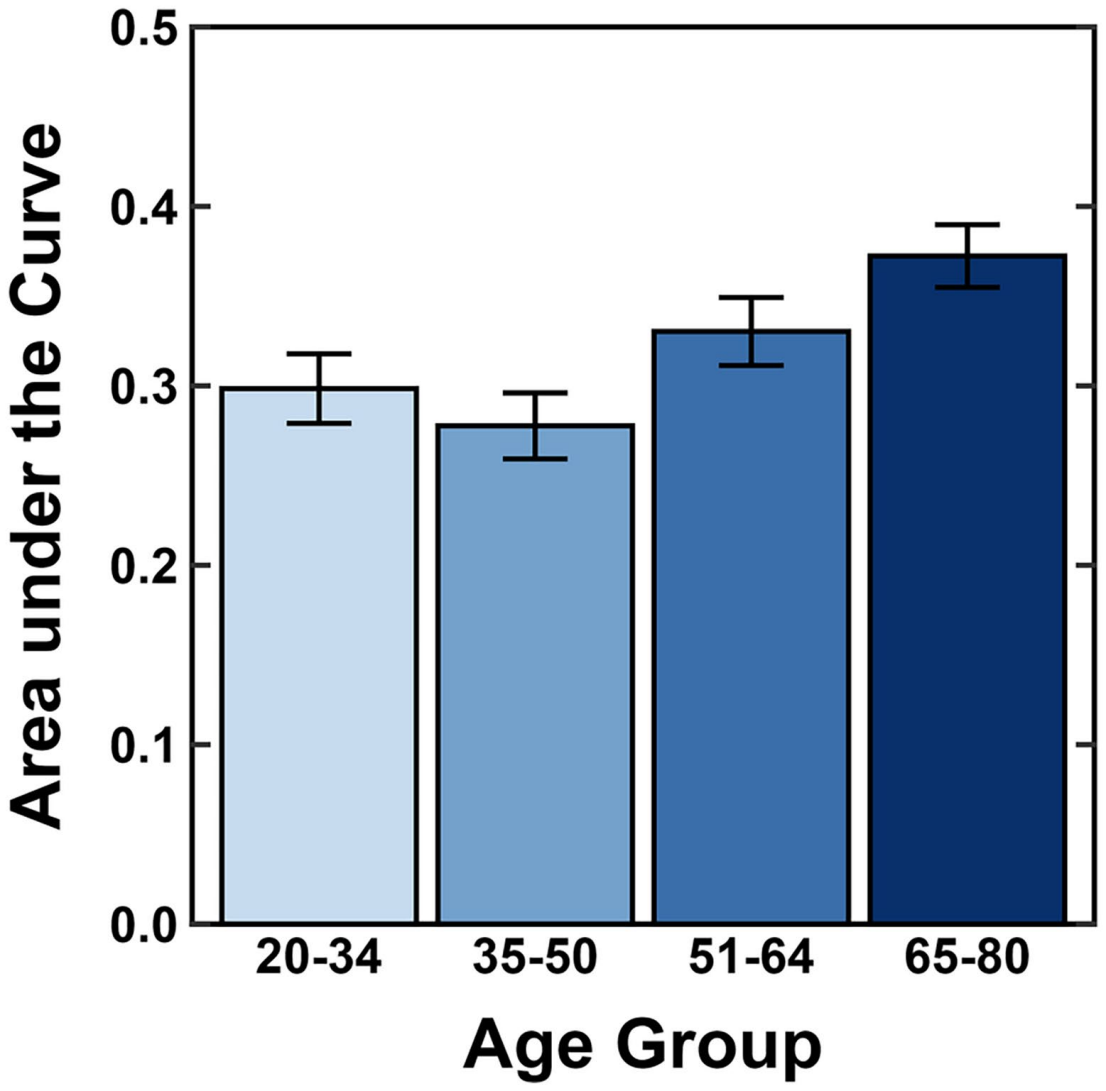
Group mean area under the curve, averaged across the three reward amounts, for each of the four age groups in Study 1. Error bars represent the standard errors of the mean.

For the sample as a whole, age was positively correlated with the mean of the AuCs of the three reward amounts, *r*(594) = 0.13, *p* = 0.002 (see Figure 2, which depicts the group mean AuCs for the four age groups shown in Figure 1). As may be seen, the older age groups had larger AuCs than the younger groups, reflecting the fact that older participants discounted delayed rewards less steeply than younger participants. As hypothesized, the correlation between income and mean AuC was not significant for participants aged 20 to 34, *r*(133) = 0.07, *p* = 0.41, but was significant for those aged 35 to 80, *r*(425) = 0.27, *p* < 0.001. Therefore, only participants aged 35 and older were included in the following analyses of the effects of age and income on discounting.

Table 2 presents the intercorrelations of age, income, education, gender, distress, anxiety, depression, health, and mean AuC for participants 35 and older. For a corresponding correlation matrix based on data from the entire sample, see Supplementary Table 1. Age was positively correlated with mean AuC, *r*(450) = 0.17, *p* < 0.001. Multilevel beta regression analyses confirmed the significant main effect of age (*p* < 0.001; see Model 1 in Table 3). We also tested for, but did not find, a significant nonlinear effect of age (i.e., the quadratic and cubic terms were not significant). As hypothesized, when income was added to the regression model (see Model 2 in Table 3), the analysis revealed that higher levels of income were associated with larger AuCs, indicating significantly shallower discounting of delayed rewards by participants with higher income levels. Note that the main effect of age remained significant across income levels.

**Table 2:** Means, standard deviations, and pairwise Pearson correlations of demographic variables, psychological distress, anxiety, depression, health, and mean area under the curve for participants aged 35–80 in Study 1.

Variable	*M*	*SD*	1	2	3	4	5	6	7	8
1. Age	56.90	12.97	–							
2. Income	2.49	1.36	−0.131**	–						
3. Education	14.44	2.34	0.005	0.343***	–					
4. Gender	0.50	0.50	0.011	−0.144**	−0.179***	–				
5. Distress	13.52	8.19	−0.308***	−0.049	−0.067	0.120*	–			
6. Anxiety	7.70	4.92	−0.349***	0.001	−0.046	0.155***	0.932***	–		
7. Depression	5.80	4.04	−0.195***	−0.103*	−0.083	0.060	0.897***	0.676***	–	
8. Health	3.15	1.00	−0.064	0.288***	0.193***	−0.146**	−0.378***	−0.260***	−0.452***	–
9. AuC	0.33	0.23	0.169***	0.269***	0.081	0.044	−0.060	−0.078	−0.031	0.076

SD = standard deviation; AuC = area under the curve. Gender was coded as 0 = Male, 1 = Female. See Supplementary Table 1 for the table for participants aged 20 to 80.

*p < 0.05, **p < 0.01, ***p < 0.001.

**Table 3:** Model estimates obtained from bootstrapping analyses that regressed discounting on age, income, psychological distress, covariates, and interactions with age for participants aged 35–80 in Study 1.

Variable	Model 1		Model 2		Model 3		Model 4	
	Est. (Est. Err.)	*p*	Est. (Est. Err.)	*p*	Est. (Est. Err.)	*p*	Est. (Est. Err.)	*p*
Age	0.490 (0.092)	**<0.001**	0.430 (0.088)	**<0.001**	0.455 (0.096)	**<0.001**	0.515 (0.109)	**<0.001**
Income			0.699 (0.088)	**<0.001**	0.663 (0.089)	**<0.001**	0.690 (0.115)	**<0.001**
Distress					0.098 (0.102)	0.340	0.138 (0.127)	0.273
Amount							0.343 (0.032)	**<0.001**
Education							0.113 (0.125)	0.387
Gender							0.262 (0.101)	**0.010**
Health							0.191 (0.117)	0.106
Age x Income							−0.507 (0.276)	0.072
Age x Distress							0.057 (0.229)	0.803
Age x Amount							−0.309 (0.070)	**<0.001**
Age x Education							0.655 (0.314)	**0.017**
Age x Gender							−0.422 (0.232)	0.070
Age x Health							−0.465 (0.239)	0.052

Est. = median of the bootstrapped coefficients; Est. Err. = standard deviation of the bootstrapped coefficients; *p* = *p*-value. Bold represents statistical significance; *p* < 0.05. Gender was coded as 0 = Male, 1 = Female. See Supplementary Table 2 for the full table of model estimates.

To assess the role of psychological distress and its components, different versions of Model 3 added either anxiety, depression, or distress (the sum of the scores on the anxiety and depression subscales), respectively, as independent variables. However, none of these effects were statistically significant (all *p* > 0.25), and the main effects of age and income remained significant with psychological distress statistically controlled (Table 3 shows the results for the version of Model 3 that added distress). Model 4 then added the independent variables amount, education, gender, distress, and health, as well as their interactions with age. The main effect of age remained significant in Model 4, as did the effect of income. Whereas Table 3 shows the interactions of all the independent variables with age, versions showing all of the two-way interactions among the independent variables, and not just the interactions with age, are shown in Supplementary Table 2.

**Figure 3:**
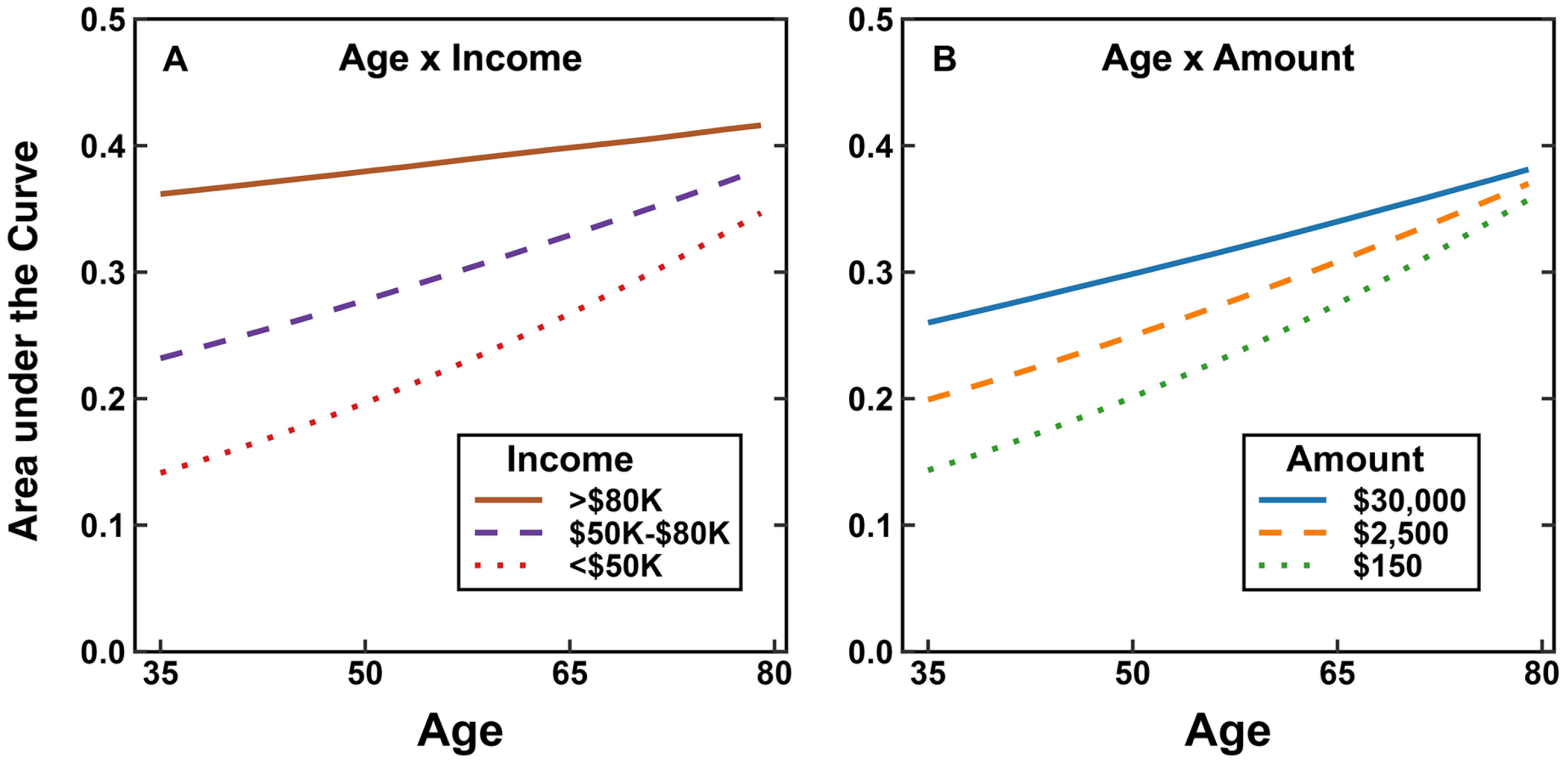
Area under the Curve as a function of age for each of the three income levels **(A)** and for the three different reward amounts **(B)** in Study 1. Lines represent the effect of age from the bootstrapping analyses, controlling for other variables at their mean levels (Model 4 in Table 3).

To assess the effects of age at each income level predicted by the buffering hypothesis, linear contrasts based on Model 4 were conducted. Results showed that whereas the effect of age was significant for the lower- and middle-income levels (both *p* < 0.001), there was no significant age effect for the higher income level (*p* = 0.543), although the interaction of income and age was not significant (see Figure 3A). Model 4 also revealed a significant interaction of age and amount: The effect of age was more pronounced at the smaller reward amounts than at the larger amounts (see Figure 3B), although linear contrasts revealed that the age effect was significant across all three delayed amounts (all *p* < 0.01). Finally, the interaction of age and education was also significant, and the effect of age increased as a function of education. Linear contrasts revealed that a significant age effect was observed only among participants with a college education or higher (*p* < 0.001), with no age effect among those with less than a college education (*p* = 0.821).

### 3.3 Discussion

The findings from Study 1 provide a robust, large-sample replication of the buffering hypothesis, confirming that the relation between age and delay discounting is moderated by income. As predicted, we observed a significant main effect of age, with older adults discounting delayed rewards less steeply than younger adults. Notably, this age-related difference was not uniform across the socioeconomic spectrum. Consistent with our previous research (Wan et al., 2024), linear contrasts revealed a pronounced age-related decrease in delay discounting among participants in the lower- and middle-income groups, but was absent among those in the highest income group. This pattern may explain inconsistencies in the discounting literature by demonstrating that age effects can be obscured when studies do not take socioeconomic status into account. Furthermore, the present finding that older adults discounted delayed rewards less steeply than younger adults challenges the predictions of theories (e.g., Socioemotional Selectivity Theory; Carstensen, 2021), that posit that older adults are more present-focused and actually discount the future more steeply than younger adults.

Our results support the core prediction of the buffering hypothesis, but they introduce important nuances. Unlike the initial study by Wan et al. (2024), in which the age difference in the lower-income group was eliminated after controlling for psychological distress, we did not find a significant predictive role for distress or its components (anxiety, depression) in the present, larger study. This divergence suggests that although distress may be a potent mediator under conditions of pronounced scarcity (as in studies with extreme income groups), its influence may be less direct in a more heterogeneous population sample in which other factors, such as wealth, debt, and subjective financial well-being, may play a larger role in decision-making.

The present results also revealed two factors, reward magnitude and education, that modulated the effects of age on discounting. The significant age x amount interaction indicated that while the age effect was present at all reward levels, it was most pronounced for the smallest reward amount ($150) and diminished as the reward size increased. This suggests that for smaller, more everyday financial decisions, the psychological maturation associated with aging may lead to more patient choices, whereas younger and older adults may discount larger, less frequently encountered amounts (e.g., $30,000) to about the same extent.

Finally, although education was not significantly correlated with AuC (Table 2), and the main effect of education was not significant after controlling for age, income, distress, amount of reward and their interactions with age, there was a significant age x education interaction (Table 3). Follow-up analyses designed to explicate this interaction revealed that with the effects of other measures and interactions statistically controlled, delay discounting decreased with age in college graduates but not among those with less education.

The observed age x education interaction is suggestive of an effect of socioeconomic status, but like other effects of SES, it is difficult to interpret due to inherent problems of multicollinearity (Jeynes, 2002; Antonoplis, 2023). Although the interaction could reflect a benefit of formal education, it could also reflect the rearing practices of college-educated parents (since their children are more likely to be future college graduates), differences in intelligence, or many other intercorrelated measures (e.g., geographical neighborhood characteristics). Future research should attempt to differentiate how these (and other) measures impact discounting. In keeping with the buffering hypothesis, this effort should begin with efforts to directly measure the psychological effects of scarcity (financial stress/well-being) that are associated with a lower income (e.g., CFPB: Consumer Financial Protection Bureau, 2017; Incharge Financial Distress/Financial Well-Being Scale: Prawitz et al., 2006).

In summary, the findings for delay discounting strongly support a model in which age-related decreases in degree of discounting are most evident among individuals with psychological and social resources associated with a high income. The results of our first study also underscore the complex interplay of age, socioeconomic status, and individual characteristics in people’s decision-making involving delayed rewards. Further, given the well-established differences between delay and probability discounting (Green and Myerson, 2004; Green et al., 2014b), the present results call for a direct comparison with the effects of age on decision-making involving probabilistic rewards. Accordingly, our second study applies a directly analogous approach to an examination of the similarities and differences in the effects of age on probability discounting.

## 4 Study 2: probability discounting

### 4.1 Method

#### 4.1.1 Participants

A sample of 664 participants was recruited for Study 2. Of these, 69 failed at least one of the six attention checks presented during the discounting part of the study, and three selected “non-binary” in response to the question regarding gender. Statistical analyses were based on the remaining 592 participants (see Table 4 for demographic information).

**Table 4:** Characteristics of participants by age group in Study 2.

Characteristics	Age group			
	20–34 (*N* = 139)	35–50 (*N* = 156)	51–64 (*N* = 122)	65–80 (*N* = 175)
Age (years), mean (SD)	27.70 (3.94)	42.51 (4.59)	57.14 (3.74)	71.61 (4.23)
Female, % (*N*)	50.36 (70)	46.79 (73)	50.00 (61)	49.14 (86)
Education (years), mean (SD)	14.26 (2.43)	14.74 (2.14)	14.24 (2.41)	14.88 (2.06)
(*N* Missing)	(1)	(1)	(0)	(0)
HADS	9.61 (4.51)	9.11 (4.78)	7.97 (4.64)	5.45 (4.22)
Anxiety, Mean (SD)	6.61 (3.91)	5.96 (4.29)	5.29 (4.12)	4.51 (3.43)
Depression, Mean (SD)	(1)	(0)	(2)	(2)
(*N* Missing)	3.50 (1.03)	3.31 (1.06)	2.97 (1.08)	3.16 (0.93)
Health, mean (SD)	(1)	(0)	(0)	(0)
(*N* Missing)	82.61 (114)	88.39 (137)	97.54 (119)	97.71 (171)
Not Hispanic/Latino, % (*N*)	(1)	(1)	(0)	(0)
(*N* Missing)	2.27 (3)	2.63 (4)	-	0.57 (1)
Race, % (*N*)	5.30 (7)	6.58 (10)	(4)	1.14 (2)
American Indian or Alaska Native	28.79 (38)	15.13 (23)	(14)	4.57 (8)
Asian or Asian American	56.06 (74)	73.03 (111)	(101)	91.43 (160)
Black or African American	7.58 (10)	2.63 (4)	(3)	1.71 (3)
Native Hawaiian or Pacific Islander	(7)	(4)	–	(1)
White	37.59 (50)	25.00 (39)	41.18 (49)	23.98 (41)
More than one race	22.56 (30)	25.64 (40)	22.69 (27)	24.56 (42)
(*N* Missing)	21.80 (29)	24.36 (38)	19.33 (23)	29.82 (51)
Income (in U. S. dollars), % (*N*)	6.77 (9)	7.69 (12)	4.20 (5)	10.53 (18)
<$30,000	11.28 (15)	17.31 (27)	12.61 (15)	11.11 (19)
$30,000–$50,000	(6)	(0)	(3)	(4)
$50,000–$80,000	27.70 (3.94)	42.51 (4.59)	57.14 (3.74)	71.61 (4.23)
$80,000–$100,000	50.36 (70)	46.79 (73)	50.00 (61)	49.14 (86)
>$100,000	14.26 (2.43)	14.74 (2.14)	14.24 (2.41)	14.88 (2.06)
(*N* Missing)	(1)	(1)	(0)	(0)

SD = standard deviation; N = number of participants; HADS = Hospital Anxiety and Depression Scale.

#### 4.1.2 Procedure

Within each of the three amount conditions, each participant made a series of six choices at each of five probabilities between a smaller, certain reward whose amount was adjusted, and a larger, probabilistic reward, whose amount remained constant within a condition. Probabilities were expressed as the percent chance of receiving the larger reward (5, 20, 50, 80%, or 95%). On each trial, if the participant chose the smaller, certain reward, its amount was decreased on the next trial, whereas if the participant chose the larger, probabilistic reward, the amount of the certain reward was increased on the next trial. The first choice in each probability condition was between the probabilistic reward and a certain reward whose amount was half that of the probabilistic reward, and the first adjustment was half of the difference between the amounts of certain and probabilistic rewards; the size of each subsequent adjustment was half that of the previous adjustment, rounded to the nearest dollar.

The adjusting-amount task converges on an amount of certain reward that approximates the subjective value (i.e., certainty equivalence) of the probabilistic reward. The amount of the certain reward that would have been presented on a seventh trial, if one had been presented, provided an estimate of the subjective value of the probabilistic reward. The procedure was repeated at each of five probabilities in order to map out a discounting function reflecting the change in the subjective value of the reward as the probability of its receipt decreased. Within each of the three amount conditions, the order of presentation of the five probability conditions was randomly determined.

### 4.2 Results

**Figure 4:**
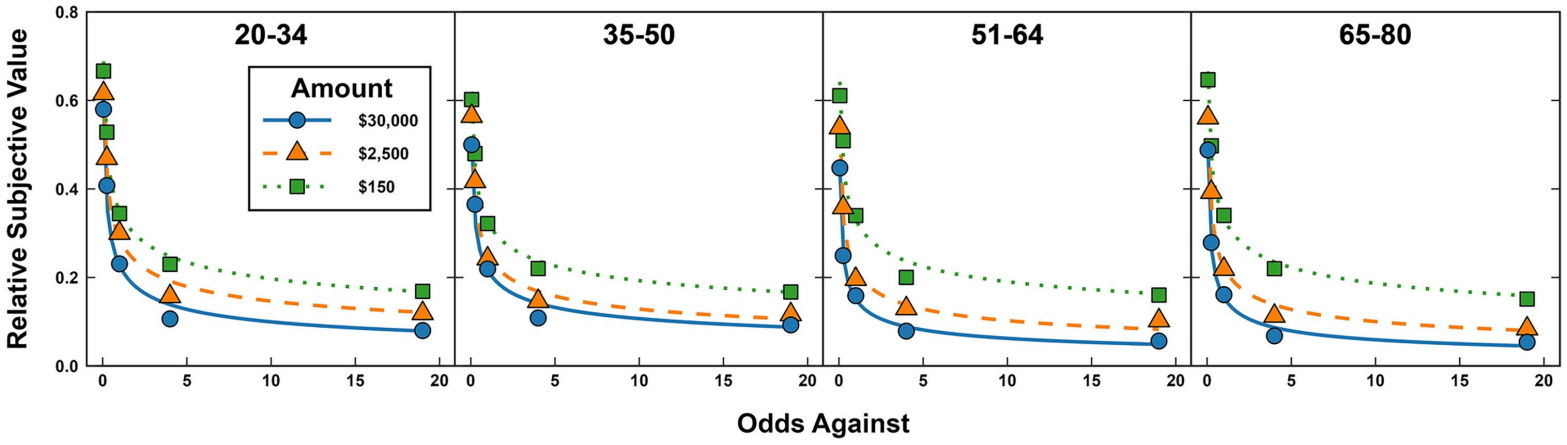
Relative subjective value of a reward as a function of the odds against its receipt for each reward amount for each age group in Study 2. Data represent the group means, and curves represent the best-fitting hyperboloid discounting functions.

Figure 4 depicts the group mean relative subjective values of the probabilistic reward (i.e., subjective value divided by the probabilistic amount) as a function of the odds against receiving the reward (i.e., 1 – the reward probability, divided by the reward probability; Rachlin et al., 1991) for each age group in each amount condition. As may be seen, systematic discounting was observed in all four age groups: Relative subjective value decreased as the odds against the reward increased in each of the three amount conditions. In each case, the hyperboloid discounting function (Myerson and Green, 1995) accounted for more than 97% of the variance. In addition, a clear reverse magnitude effect was observed, that is, larger probabilistic amounts were discounted more steeply than smaller probabilistic amounts. For all four groups, linear contrasts based on beta regressions verified that there was a systematic decrease in AuC as a function of amount (all *p* < 0.001).

**Figure 5:**
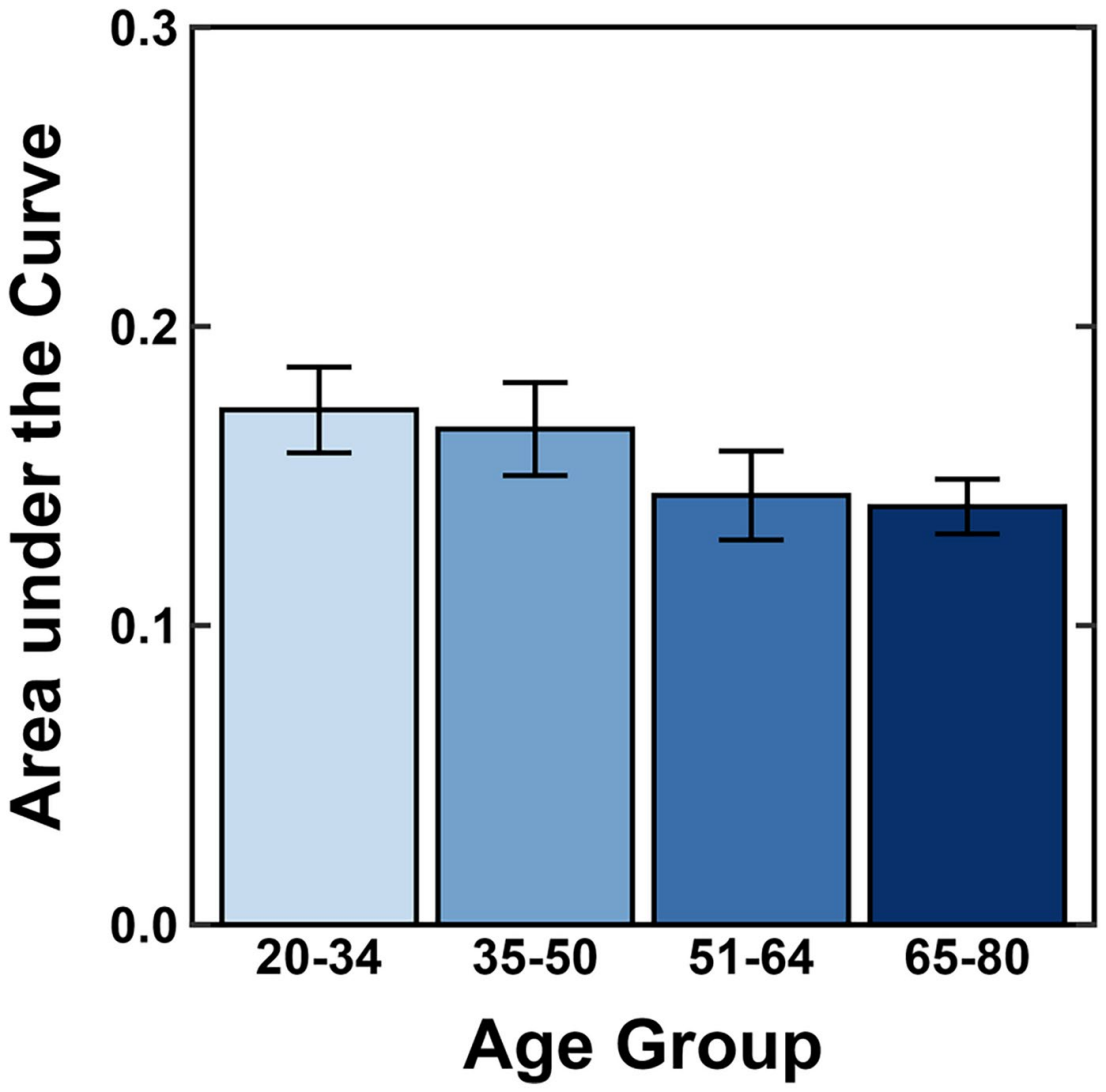
Group mean Area under the Curve, averaged across the three reward amounts, for each of the four age groups in Study 2. Error bars represent the standard errors of the mean.

For the sample as a whole, age was negatively correlated with the mean AuC of the three reward amounts, *r*(590) = −0.10, *p* = 0.013 (see Figure 5, which depicts the group mean AuCs for the four age groups depicted in Figure 4). As may be seen, the older age groups had smaller AuCs than the younger groups, reflecting the fact that older participants were more risk-averse than younger participants. As in Study 1, we examined the effects of income on discounting in those under 35 separately. The correlation between income and mean AuC was not significant for either participants aged 20 to 34 or participants aged 35–80 (both *p* > 0.08).

**Figure 6:**
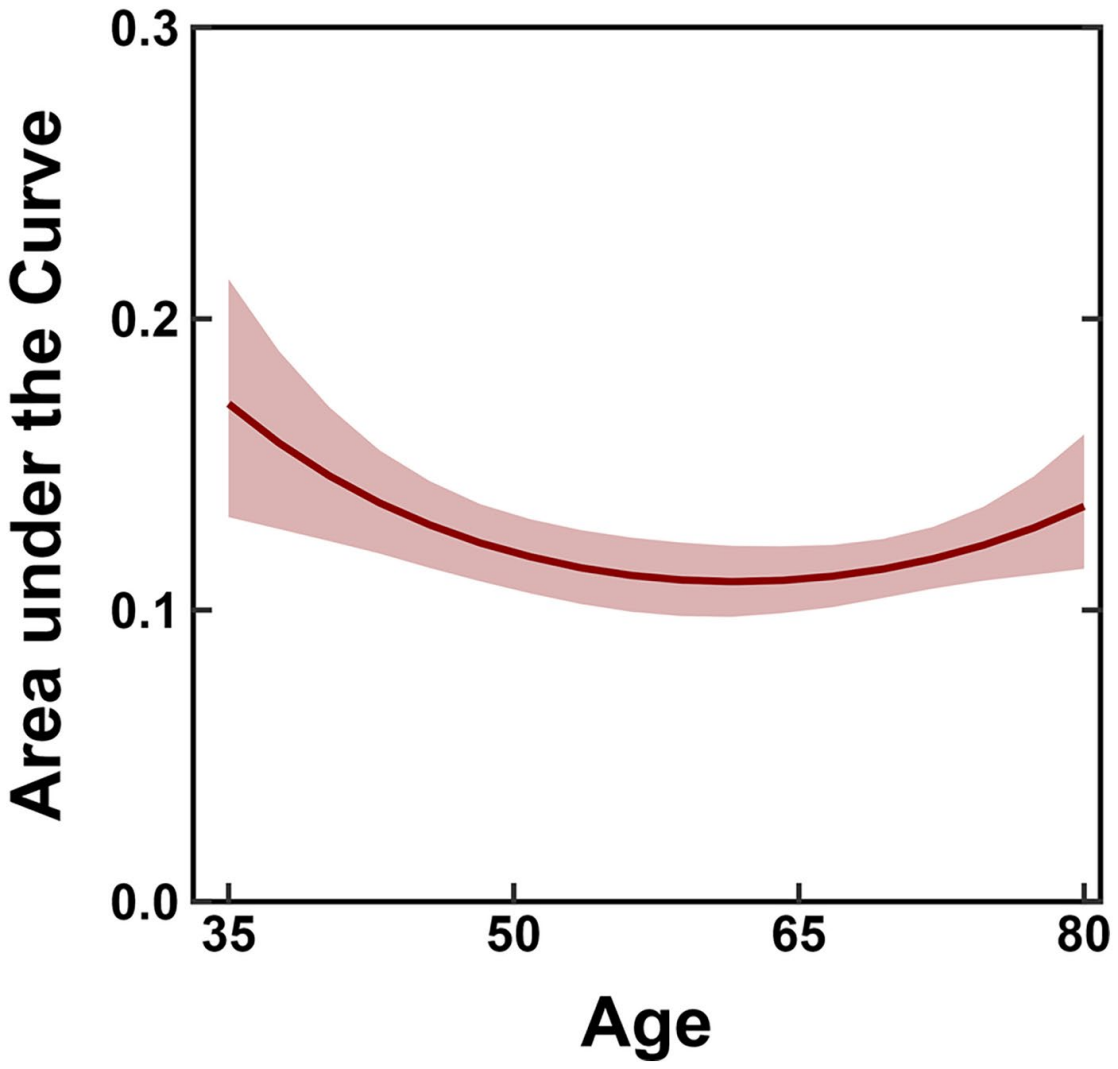
Area under the Curve as a function of age in Study 2. The curve represents the quadratic effect of age from the bootstrapping analyses, and the shaded region represents the 95% confidence interval for the regression line (Model 1 in Table 6).

Table 5 presents the intercorrelations of age, income, education, gender, distress, anxiety, depression, health, and mean AuC for participants 35 and older. For a corresponding correlation matrix based on data from the entire sample, see Supplementary Table 3. A simple correlation between age and mean AuC was significant, *r*(451) = −0.10, *p* = 0.040. However, beta regression analyses revealed that the relation between age and AuC was nonlinear (i.e., the quadratic term was significant, *p* = 0.011; see Model 1 in Table 6), reflecting an increase in risk aversion between young adulthood and middle age with relatively little change in discounting thereafter (see Figure 6). Consistent with the correlation analysis, adding income to the regression model failed to reveal significant income effects (see Models 2–4 in Table 6). Note that the quadratic effect of age on AuC remained significant.

**Table 5:** Means, standard deviations, and pairwise Pearson correlations of demographic variables, psychological distress, anxiety, depression, health, and mean area under the curve for participants aged 35–80 in Study 2.

Variable	*M*	*SD*	1	2	3	4	5	6	7	8
1. Age	57.69	13.13	–							
2. Income	2.53	1.35	−0.037	–						
3. Education	14.66	2.20	0.015	0.327***	–					
4. Gender	0.49	0.50	0.039	−0.176***	−0.111*	–				
5. Distress	12.63	7.87	−0.266***	−0.112*	−0.111*	0.047	–			
6. Anxiety	7.40	4.80	−0.327***	−0.045	−0.111*	0.100*	0.916***	–		
7. Depression	5.22	3.97	−0.134**	−0.169***	−0.080	−0.033	0.874***	0.606***	–	
8. Health	3.16	1.02	−0.079	0.289***	0.082	−0.052	−0.396***	−0.261***	−0.471***	–
9. AuC	0.15	0.16	−0.096*	0.039	−0.040	−0.025	0.088	0.143**	−0.002	0.100*

SD = standard deviation; AuC = area under the curve. Gender was coded as 0 = Male, 1 = Female. See Supplementary Table 3 for the table for participants aged 20–80.

*p < 0.05, **p < 0.01, ***p < 0.001.

**Table 6:** Model estimates obtained from bootstrapping analyses that regressed discounting on age, income, anxiety, covariates, and interactions with age for participants aged 35–80 in Study 2.

Variable	Model 1		Model 2		Model 3		Model 4	
	Est. (Est. Err.)	*p*	Est. (Est. Err.)	*p*	Est. (Est. Err.)	*p*	Est. (Est. Err.)	*p*
Age	−0.142 (0.080)	0.075	−0.131 (0.081)	0.113	−0.052 (0.089)	0.568	0.073 (0.230)	0.739
Age^2^	0.505 (0.192)	**0.011**	0.483 (0.200)	**0.015**	0.238 (0.105)	**0.010**	0.544 (0.253)	**0.034**
Income			0.141 (0.082)	0.094	0.519 (0.201)	0.058	0.307 (0.168)	0.071
Anxiety					0.157 (0.082)	**0.022**	0.426 (0.200)	0.054
Amount							−1.103 (0.059)	**<0.001**
Education							−0.257 (0.158)	0.118
Gender							−0.118 (0.155)	0.454
Health							0.268 (0.193)	0.177
Age x Income							−0.164 (0.245)	0.502
Age x Anxiety							−0.110 (0.248)	0.665
Age x Amount							−0.301 (0.087)	**0.001**
Age x Education							0.019 (0.276)	0.940
Age x Gender							−0.101 (0.234)	0.673
Age x Health							0.192 (0.253)	0.429
Age^2^ x Income							−0.899 (0.509)	0.094
Age^2^ x Anxiety							−0.450 (0.660)	0.518
Age^2^ x Amount							0.431 (0.177)	**0.019**
Age^2^ x Education							0.296 (0.488)	0.539
Age^2^ x Gender							−0.479 (0.500)	0.327
Age^2^ x Health							0.064 (0.571)	0.909

Est. = median of the bootstrapped coefficients; Est. Err. = standard deviation of the bootstrapped coefficients; *p* = *p*-value. Bold represents statistical significance; *p* < 0.05. Gender was coded as 0 = Male, 1 = Female. See Supplementary Table 4 for the full table of model estimates.

To assess the role of psychological distress and its components, different versions of Model 3 added either anxiety, depression, or distress, respectively, as independent variables. Because only anxiety was significant, Table 6 shows the results for the version of Model 3 that added anxiety; note that the quadratic effect of age remained significant. Model 4 then added the independent variables amount, education, gender, and health, as well as the interactions of all the variables with age. The quadratic effect of age continued to be significant in Model 4, as did the main effect of anxiety. Supplementary Table 4 presents the results for all of the two-way interactions among the independent variables.

**Figure 7:**
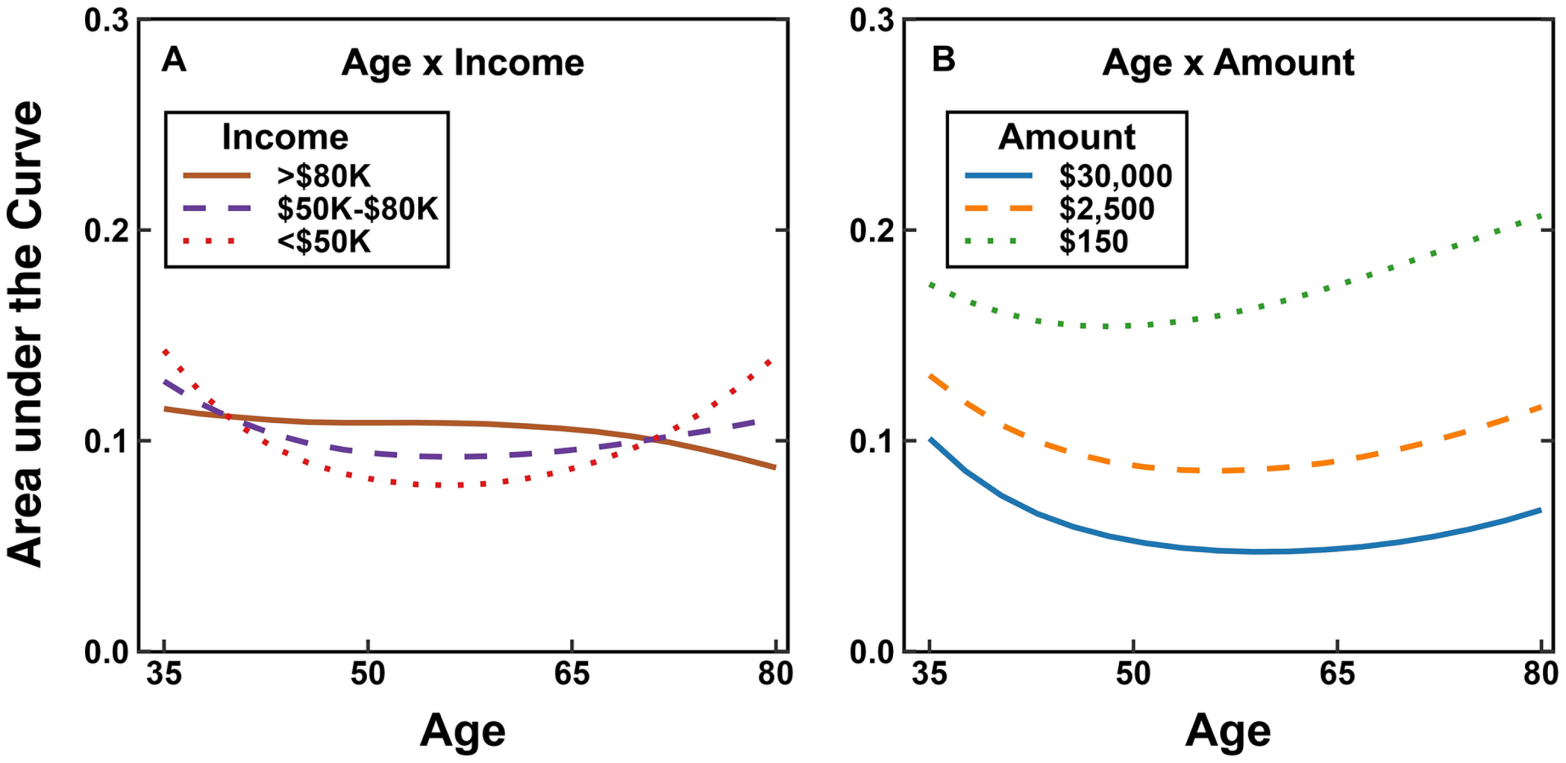
Area under the Curve as a function of age for each of the three income levels **(A)** and for the three different reward amounts **(B)** in Study 2. The curve represents the effect of age from the bootstrapping analyses, controlling for other variables at their mean levels (Model 4 in Table 6).

To assess the effects of age at each income level, linear contrasts based on Model 4 were also conducted. Consistent with the buffering hypothesis, the quadratic effect of age was significant for the lowest-income level (*p* = 0.012), but not for the middle- and higher-income levels (both *p* > 0.14). Indeed, although the interactions of income with age and with age^2^ were not significant, the quadratic effect decreased as income increased, and AuC remained virtually constant with age in the highest income group (see Figure 7A). Finally, the interactions of age and amount and of age^2^ and amount were significant (see Figure 7B), indicating that the influence of age on risky choice was modulated by the amount of the potential reward.

### 4.3 Discussion

In Study 2, consistent with previous studies of probability discounting (e.g., Du et al., 2002; Green et al., 1999; Rachlin et al., 2000), larger probabilistic rewards were discounted more steeply than smaller rewards by both younger and older adults. This effect is the opposite of what was observed with delayed rewards in Study 1. More importantly, older adults overall were more risk-averse than younger adults, although the relation between age and degree of discounting was nonlinear (i.e., age^2^ was a significant predictor of AuC): Discounting was shallowest from young adult to early middle age before stabilizing in later life.

Moreover, the data were consistent with the buffering hypothesis in that although the effect of age did not decrease with income, the quadratic effect of age was only significant for the lowest income group, and age had virtually no effect on discounting in the highest income group. This suggests that a high income may act as a buffer in that it affords individuals a stable level of risk tolerance across the lifespan regardless of nonfinancial changes in their circumstances. This significant nonlinear effect may explain the inconsistent findings in studies of age and risky choice (see Mata et al., 2011; Best and Charness, 2015; Bagaïni et al., 2023).

## 5 General discussion

In order to facilitate comparisons of the effects of delay and probability on the subjective value of a choice outcome (i.e., its present and/or certain equivalent), the current study applied a unified discounting framework using analogous models and assessment procedures (Du et al., 2002; Green and Myerson, 2004). The present results demonstrate that age-related differences in the valuation of delayed and probabilistic rewards follow different trajectories. By evaluating both domains within this standardized framework, the observed differences in what economists refer to as intertemporal choice and risky choice can be reliably attributed to domain-specific valuation processes, rather than to methodological confounds or a generalized shift in reward sensitivity. These results address inconsistencies in the aging literature by confirming that age-related changes in economic preferences are systematically moderated by socioeconomic context.

Recently, Wan et al. (2024) proposed a buffering hypothesis to explain the effects of income on age differences in the discounting of delayed rewards. Applying the discounting framework, they observed that although there were age differences between adults with lower incomes, no age differences were observed between those with higher incomes. They attributed this effect to age-related increases in emotional stability (Terracciano et al., 2005; Costa et al., 2019) that buffer older adults against various stressors, including financial scarcity. The buffering hypothesis predicts that although financial scarcity (i.e., a low income) will lead to steep discounting in younger adults, it will have less effect on older adults, leading to a significant age difference. In contrast, the absence of financial scarcity (i.e., high income) is not associated with psychological distress, and thus both high-income younger and older adults will show shallower discounting, and the size of the age-related difference will decrease, as was observed in both of the present studies.

As expected, income was unrelated to the degree of both delay and probability discounting in both Study 1 (with delayed rewards) and Study 2 (with probabilistic rewards) by participants younger than 35 because their self-reported income is not a reliable indicator of their experienced financial scarcity due to parental financial support (US Census Bureau, 2023). For those 35 and older, there were two pronounced differences between the effects of age, income, and reward amount on delay and probability discounting. First, delay discounting decreased linearly with age in Study 1. In contrast, Study 2 revealed a nonlinear relation between discounting and age such that, averaged across income levels, discounting increased (i.e., more risk averse) between age 35 and middle age, with relatively little change thereafter. Notably, however, the nonlinearity was not observed in high income participants, who showed a relatively constant level of discounting regardless of age. Second, consistent with previous research (e.g., Green et al., 1999; Myerson et al., 2011), larger amounts of delayed reward were discounted less steeply than smaller amounts, whereas larger amounts of probabilistic reward were discounted more steeply, and these findings were observed in all groups of participants, regardless of age and income.

The Wan et al. (2024) study reported that the age effect on delay discounting was eliminated after statistically controlling for psychological distress, although this finding was not replicated in Study 1. Moreover, distress, at least in the form of anxiety, had a significant effect on probability discounting in Study 2, although when distress (anxiety) was statistically controlled, the quadratic effect of age remained significant. Importantly, the age-related decrease in degree of discounting was present only among lower- and middle-income participants, with no significant age effects observed in the high-income group, consistent with the buffering hypothesis.

In contrast to the lower- and middle-income groups, the high-income group displayed a relatively stable level of risk preference across the lifespan, consistent with the hypothesis that income does exert a buffering effect, albeit one with a different mechanism than for delay discounting. Rather than mitigating scarcity-induced psychological stress, high income may provide a stable resource base that insulates decision-making from the age-related psychological shifts that appear to drive changes in risk preferences for those with fewer resources. Unlike for delay discounting in Study 1, psychological distress (i.e., higher anxiety) was a significant predictor of greater risk aversion in Study 2, independent of age and income. This pattern of findings – a nonlinear age effect that is itself moderated by income and independently influenced by anxiety – helps to explain the lack of a simple, robust age effect in prior meta-analyses of risky choice.

Although the present findings indicate the moderating role of income on age-related discounting trajectories, income serves as an objective proxy for financial scarcity. The buffering hypothesis posits that financial scarcity induces psychological distress, which subsequently influences degree of discounting. We assessed psychological distress (e.g., state anxiety) in the current study and found that it independently predicted risky choice. Nevertheless, a general distress scale does not isolate the specific, acute experience of financial precarity. Furthermore, the observed moderation by income may capture variance related to structural assets rather than reflecting the buffering of emotional distress. Income and accumulated wealth covary with objective competencies, such as financial literacy, and the choice patterns observed in higher-income older adults, such as shallower delay discounting and increased risk aversion, could result from the application of their acquired financial knowledge. As a consequence, accumulated resources and objective literacy act as a threshold enabling context-dependent valuation strategies, such as prioritizing wealth accumulation in intertemporal choices or asset preservation in risky choices. To determine whether age-related differences in discounting result primarily from an increase in emotional buffering or from the accumulation of structural resources, subsequent investigations should integrate validated, direct measures of subjective financial stress and objective financial literacy. Instruments such as the Consumer Financial Protection Bureau (CFPB) Financial Well-Being Scale (Consumer Financial Protection Bureau, 2017) and the InCharge Financial Distress Scale (Prawitz et al., 2006) provide assessments of the subjective experience of financial scarcity. Incorporating such measures may clarify how specific dimensions of financial stress and financial literacy influence valuation in both intertemporal and risky choice.

Although the buffering hypothesis was supported with respect to the discounting of delayed gains, recent work from our laboratory has shown that a different pattern emerges with delayed monetary losses, with significant age-related differences in discounting appearing only at higher income levels (Wan et al., 2025). This divergence suggests that the interplay of age and income may not be uniform across different decision domains and underscores the need for further investigations in the risky choice domain.

Notably, the findings from both studies are inconsistent with predictions derived from Socioemotional Selectivity Theory (Carstensen, 2006). A central tenet of Socioemotional Selectivity Theory is that shrinking future time horizons cause older adults to prioritize present-oriented goals, which predicts that they should devalue future rewards more heavily and thus exhibit steeper delay discounting. Our finding of a decrease in the degree of delay discounting with age is in direct opposition to this prediction. Furthermore, the comparison of preferences across different life stages may warrant caution. For example, Caliendo and Findley (2014) demonstrated that comparing discount functions within finite-horizon models needs to adjust for the diminishing temporal horizon to maintain a constant overall level of impatience; without such an adjustment, differences in intertemporal choice might be incorrectly attributed to psychological shifts in self-control rather than to the methodological artifact of evaluating a shorter future. It should be noted that our empirical findings are consistent with recent large-scale meta-analyses that also failed to find evidence for increased discounting in later life (Bagaïni et al., 2023; Seaman et al., 2022). The results for risky choice do provide at least some support for the predictions of Socioemotional Selectivity Theory. That is, the theory predicts a “positivity effect,” in which older adults prioritize positive emotional information (Carstensen and DeLiema, 2018). This might lead to their becoming less risk-averse when focusing on potential gains. Our finding of a nonlinear trajectory with a slight decrease in risk aversion (i.e., increases in AuCs) after middle age provides some support for this interpretation.

A potential limitation of the present research is the reliance on hypothetical rather than real monetary rewards, which raises questions of ecological validity. Hypothetical choice paradigms introduce the risk that stated preferences may deviate from actual behavior involving real financial stakes. However, investigating the magnitude effect across a wider range of values (e.g., $2,500 and $30,000 as in the present study) would not be feasible as actual rewards in experimental studies. Notably, the results from a number of empirical studies indicate consistent correspondence between hypothetical and real choices in discounting paradigms: Within-subject comparisons demonstrate that the discounting of both real and hypothetical rewards is well-described by similar hyperbolic functions, and comparison of choices involving the two reward types has failed to reveal significant differences in the steepness of discounting (Johnson and Bickel, 2002; Madden et al., 2003). Furthermore, using small, real monetary rewards introduces alternative threats to ecological validity; providing immediate cash often shifts the laboratory decision-making context toward strict monetary maximization, which may fail to capture the everyday valuation processes associated with larger financial decisions (Locey et al., 2011). Consequently, the existing literature supports the validity of using hypothetical rewards to examine choice behavior, particularly when evaluating variations across large reward magnitudes.

Another concern of the present research is its use of a cross-sectional design, which necessarily conflates intra-individual aging effects with inter-individual cohort differences. Although the observed age-related trajectories in both delay and probability discounting – and their moderation by income – are consistent with the psychological processes posited by the buffering hypothesis, such as age-related increases in emotional stability, these patterns could alternatively reflect systematic generational variances in economic socialization, historical financial conditions, or cumulative wealth accumulation patterns unique to specific cohorts. For example, older adults may show specific risk tolerances or intertemporal preferences contingent upon the macroeconomic conditions prevalent during their formative years. Consequently, while the present data identify age-by-income interactions, longitudinal investigations would be necessary to distinguish the ontogenetic processes of aging from cohort-specific economic experiences.

In conclusion, the present research challenges a monolithic view of how aging affects financial preferences by demonstrating that age-related trends are domain-specific. Indeed, although preferences are affected by the socioeconomic context and the amounts of the rewards involved in both the delay discounting and probability discounting domains, they are affected in different ways. Our findings suggest that willingness to wait for a larger delayed reward increases significantly with age, but that this effect is primarily observed in lower-income individuals, consistent with the buffering hypothesis. In contrast, risk aversion follows a different trajectory, increasing from young adulthood until middle age and slightly increasing thereafter, an effect that is also moderated by income.

These divergent patterns are not well-explained by broad theories like Socioemotional Selectivity Theory but instead point to a more nuanced reality where the psychological processes of aging interact with an individual’s life circumstances. The findings for delay discounting are encouraging, suggesting that the quality of intertemporal decisions may improve as individuals age and appear to become more patient. The findings for risky choice, however, highlight a complex shift toward caution in later life that could be either adaptive or maladaptive. Ultimately, our results underscore the critical importance of moving beyond a one-size-fits-all approach, arguing instead that a context-dependent perspective is essential for understanding decision-making across the adult lifespan.

## Data Availability

The datasets presented in this study can be found in online repositories. The original contributions presented in the study are included in the article/Supplementary material. Further inquiries can be directed to the corresponding author.
