# Supplementary material for: Age, income, and the discounting of delayed and probabilistic rewards
Source: Front Psychol. 2026 Jun 12;17:1765142. doi: 10.3389/fpsyg.2026.1765142 (PMC13308517; doi:10.3389/fpsyg.2026.1765142)
Supplement: Supplementary file 2 [file Supplementary_Table_1.DOCX]

***Supplementary Material***

**Supplementary Table 1.** Means, standard deviations, and pairwise Pearson correlations of demographic variables, psychological distress, anxiety, depression, health, and mean Area under the Curve for participants aged 20 to 80 in Study 1.

| **Variable** | ***M*** | ***SD*** | **1** | **2** | **3** | **4** | **5** | **6** | **7** | **8** |
| --- | --- | --- | --- | --- | --- | --- | --- | --- | --- | --- |
| **1. Age** | 49.69 | 17.20 | - |  |  |  |  |  |  |  |
| **2. Income** | 2.50 | 1.36 | -.078 | - |  |  |  |  |  |  |
| **3. Education** | 14.33 | 2.48 | .078 | .370*** | - |  |  |  |  |  |
| **4. Gender** | 0.50 | 0.50 | .008 | -.192*** | -.152*** | - |  |  |  |  |
| **5. Distress** | 14.45 | 8.19 | -.339*** | -0.040 | -.118** | .074 | - |  |  |  |
| **6. Anxiety** | 8.43 | 5.03 | -.396*** | .001 | -.100* | .100* | .929*** | - |  |  |
| **7. Depression** | 6.03 | 3.99 | -.201*** | -.084* | -.114** | .032 | .885*** | .650*** | - |  |
| **8. Health** | 3.23 | 1.02 | -.136*** | .277*** | .162*** | -.169*** | -.330*** | -.191*** | -.430*** | - |
| **9. AuC** | 0.32 | 0.23 | .126** | .219*** | .068 | .008 | -.064 | -.066 | -.054 | .091* |

*Note.* SD = standard deviation; AuC = area under the curve; Gender was coded as 0 = male, 1 = female.

** p* < .05; *** p* < .01; **** p* < .001

**Supplementary Table 2.** Model estimates obtained from bootstrapping analyses that regressed discounting on age, income, psychological distress, covariates, and all two-way interactions for participants aged 35 to 80 in Study 1.

|  | **Model 1** | | **Model 2** | | **Model 3** | | **Model 4** | |
| --- | --- | --- | --- | --- | --- | --- | --- | --- |
|  | Est. (Est. Err.) | *p* | Est. (Est. Err.) | *p* | Est. (Est. Err.) | *p* | Est. (Est. Err.) | *p* |
| (Intercept) | -.908 (.047) | <.001 | -.885 (.045) | <.001 | -.882 (.045) | <.001 | -.966 (.056) | <.001 |
| **Age** | .490 (.092) | **<.001** | .430 (.088) | **<.001** | .455 (.096) | **<.001** | .515 (.109) | **<.001** |
| **Income** |  |  | .699 (.088) | **<.001** | .663 (.089) | **<.001** | .690 (.115) | **<.001** |
| Distress |  |  |  |  | .098 (.102) | .340 | .138 (.127) | .273 |
| **Amount** |  |  |  |  |  |  | .343 (.032) | **<.001** |
| Education |  |  |  |  |  |  | .113 (.125) | .387 |
| **Gender** |  |  |  |  |  |  | .262 (.101) | **.010** |
| Health |  |  |  |  |  |  | .191 (.117) | .106 |
| Age x Income | |  |  |  |  |  | -.507 (.276) | .072 |
| Age x Distress | |  |  |  |  |  | .057 (.229) | .803 |
| **Age x Amount** | |  |  |  |  |  | -.309 (.070) | **<.001** |
| **Age x Education** | |  |  |  |  |  | .655 (.314) | **.017** |
| Age x Gender | |  |  |  |  |  | -.422 (.232) | .070 |
| Age x Health | |  |  |  |  |  | -.465 (.239) | .052 |
| Income x Distress | |  |  |  |  |  | -.484 (.270) | .071 |
| **Income x Amount** | |  |  |  |  |  | .184 (.078) | **.021** |
| Income x Education | |  |  |  |  |  | -.129 (.286) | .627 |
| Income x Gender | |  |  |  |  |  | .025 (.221) | .906 |
| Income x Health | |  |  |  |  |  | -.214 (.254) | .400 |
| **Distress x Amount** | |  |  |  |  |  | -.208 (.076) | **.007** |
| **Distress x Education** | |  |  |  |  |  | .931 (.312) | **.002** |
| Distress x Gender | |  |  |  |  |  | .387 (.248) | .115 |
| Distress x Health | |  |  |  |  |  | -.280 (.248) | .254 |
| Amount x Education | |  |  |  |  |  | -.180 (.099) | .068 |
| **Amount x Gender** | |  |  |  |  |  | .162 (.064) | **.010** |
| Amount x Health | |  |  |  |  |  | .087 (.080) | .260 |
| Education x Gender | |  |  |  |  |  | .014 (.249) | .966 |
| Education x Health | |  |  |  |  |  | .281 (.335) | .388 |
| Gender x Health | |  |  |  |  |  | .170 (.227) | .447 |

*Note. Est.* = median of the bootstrapped coefficients; *Est. Err.* = standard deviation of the bootstrapped coefficients; *p* = *p*-value. (Bold represents statistical significance; *p* < .05.); Gender was coded as 0 = male, 1 = female.

**Supplementary Table 3.** Means, standard deviations, and pairwise Pearson correlations of demographic variables, psychological distress, anxiety, depression, health, and mean Area under the Curve for participants aged 20 to 80 in Study 2.

| **Variable** | ***M*** | ***SD*** | **1** | **2** | **3** | **4** | **5** | **6** | **7** | **8** |
| --- | --- | --- | --- | --- | --- | --- | --- | --- | --- | --- |
| **1. Age** | 50.65 | 17.25 | - |  |  |  |  |  |  |  |
| **2. Income** | 2.48 | 1.35 | .039 | - |  |  |  |  |  |  |
| **3. Education** | 14.56 | 2.26 | .069 | .329*** | - |  |  |  |  |  |
| **4. Gender** | 0.49 | 0.50 | .013 | -.150*** | -.114** | - |  |  |  |  |
| **5. Distress** | 13.47 | 7.97 | -.294*** | -.147*** | -.155*** | .053 | - |  |  |  |
| **6. Anxiety** | 7.91 | 4.82 | -.336*** | -.090* | -.167*** | .103* | .921*** | - |  |  |
| **7. Depression** | 5.55 | 4.00 | -.183*** | -.185*** | -.104* | -.022 | .883*** | .630*** | - |  |
| **8. Health** | 3.24 | 1.03 | -.158*** | .252*** | .072 | -.030 | -.361*** | -.240*** | -.431*** | - |
| **9. AuC** | 0.15 | 0.16 | -.102* | .045 | -.021 | .003 | .088* | .147*** | -.003 | .120** |

*Note.* SD = standard deviation; AuC = area under the curve; Gender was coded as 0 = male, 1 = female.

** p* < .05; *** p* < .01; **** p* < .001

**Supplementary Table 4.** Model estimates obtained from bootstrapping analyses that regressed discounting on age, income, anxiety, covariates, and all two-way interactions for participants aged 35 to 80 in Study 2.

|  | **Model 1** | | **Model 2** | | **Model 3** | | **Model 4** | |
| --- | --- | --- | --- | --- | --- | --- | --- | --- |
|  | Est. (Est. Err.) | *p* | Est. (Est. Err.) | *p* | Est. (Est. Err.) | *p* | Est. (Est. Err.) | *p* |
| (Intercept) | -2.083 (.065) | <.001 | -2.071 (.067) | <.001 | -2.085 (.067) | <.001 | -2.337 (.089) | <.001 |
| Age | -.142 (.080) | .075 | -.131 (.081) | .113 | -.052 (.089) | .568 | .073 (.230) | .739 |
| **Age^2^** | .505 (.192) | **.011** | .483 (.200) | **.015** | .519 (.201) | **.010** | .544 (.253) | **.034** |
| **Income** |  |  | .141 (.082) | .094 | .157 (.082) | .058 | .307 (.168) | .071 |
| **Anxiety** |  |  |  |  | .238 (.105) | **.022** | .426 (.200) | .054 |
| **Amount** |  |  |  |  |  |  | -1.104 (.059) | **<.001** |
| Education |  |  |  |  |  |  | -.257 (.158) | .118 |
| Gender |  |  |  |  |  |  | -.118 (.155) | .454 |
| Health |  |  |  |  |  |  | .268 (.193) | .177 |
| Age x Income | |  |  |  |  |  | -.164 (.245) | .502 |
| Age x Anxiety | |  |  |  |  |  | -.110 (.248) | .665 |
| **Age x Amount** | |  |  |  |  |  | -.301 (.087) | **.001** |
| Age x Education | |  |  |  |  |  | .019 (.276) | .940 |
| Age x Gender | |  |  |  |  |  | -.101 (.234) | .673 |
| Age x Health | |  |  |  |  |  | .192 (.253) | .429 |
| Age x Age^2^ | |  |  |  |  |  | -.187 (.524) | .712 |
| Age^2^ x Income | |  |  |  |  |  | -.899 (.509) | .094 |
| Age^2^ x Anxiety | |  |  |  |  |  | -.450 (.660) | .518 |
| **Age^2^ x Amount** | |  |  |  |  |  | .431 (.177) | **.019** |
| Age^2^ x Education | |  |  |  |  |  | .296 (.488) | .539 |
| Age^2^ x Gender | |  |  |  |  |  | -.479 (.500) | .327 |
| Age^2^ x Health | |  |  |  |  |  | .064 (.571) | .909 |
| Income x Anxiety | |  |  |  |  |  | .657 (.323) | .058 |
| Income x Amount | |  |  |  |  |  | -.092 (.077) | .241 |
| Income x Education | |  |  |  |  |  | .139 (.218) | .504 |
| Income x Gender | |  |  |  |  |  | -.310 (.228) | .181 |
| Income x Health | |  |  |  |  |  | .210 (.252) | .401 |
| **Anxiety x Amount** | |  |  |  |  |  | -.169 (.086) | **.047** |
| **Anxiety x Education** | |  |  |  |  |  | -.779 (.345) | **.024** |
| Anxiety x Gender | |  |  |  |  |  | -.244 (.271) | .370 |
| Anxiety x Health | |  |  |  |  |  | .151 (.267) | .568 |
| Amount x Education | |  |  |  |  |  | .029 (.071) | .672 |
| Amount x Gender | |  |  |  |  |  | .005 (.072) | .944 |
| Amount x Health | |  |  |  |  |  | .100 (.074) | .176 |
| Education x Gender | |  |  |  |  |  | .312 (.250) | .228 |
| Education x Health | |  |  |  |  |  | -.285 (.246) | .213 |
| Gender x Health | |  |  |  |  |  | -.020 (.232) | .931 |

*Note.* Est. = median of the bootstrapped coefficients; Est. Err. = standard deviation of the bootstrapped coefficients; *p* = *p*-value. (Bold represents statistical significance; *p* < .05.); Gender was coded as 0 = male, 1 = female.
